# Structural progression of amyloid-β Arctic mutant aggregation in cells revealed by multiparametric imaging

**DOI:** 10.1074/jbc.RA118.004511

**Published:** 2018-11-30

**Authors:** Meng Lu, Neil Williamson, Ajay Mishra, Claire H. Michel, Clemens F. Kaminski, Alan Tunnacliffe, Gabriele S. Kaminski Schierle

**Affiliations:** From the ‡ Cambridge Infinitus Research Centre, Department of Chemical Engineering and Biotechnology, University of Cambridge, Cambridge CB3 0AS, United Kingdom and; § Department of Chemical Engineering and Biotechnology, University of Cambridge, West Cambridge Site, Philippa Fawcett Drive, Cambridge CB3 0AS, United Kingdom

**Keywords:** protein aggregation, amyloid-β (Aβ), molecular dynamics, microscopic imaging, structural model, 3D structure of amyloid aggregates, Alzheimer's disease, amyloid-β, Arctic mutant, neurodegeneration, SIM

## Abstract

The 42-amino-acid β-amyloid (Aβ42) is a critical causative agent in the pathology of Alzheimer's disease. The hereditary Arctic mutation of Aβ42 (E22G) leads to increased intracellular accumulation of β-amyloid in early-onset Alzheimer's disease. However, it remains largely unknown how the Arctic mutant variant leads to aggressive protein aggregation and increased intracellular toxicity. Here, we constructed stable cell lines expressing fluorescent-tagged wildtype (WT) and E22G Aβ42 to study the aggregation kinetics of the Arctic Aβ42 mutant peptide and its heterogeneous structural forms. Arctic-mutant peptides assemble and form fibrils at a much faster rate than WT peptides. We identified five categories of intracellular aggregate—oligomers, single fibrils, fibril bundles, clusters, and aggresomes—that underline the heterogeneity of these Aβ42 aggregates and represent the progression of Aβ42 aggregation within the cell. Fluorescence-lifetime imaging (FLIM) and 3D structural illumination microscopy (SIM) showed that all aggregate species displayed highly compact structures with strong affinity between individual fibrils. We also found that aggregates formed by Arctic mutant Aβ42 were more resistant to intracellular degradation than their WT counterparts. Our findings uncover the structural basis of the progression of Arctic mutant Aβ42 aggregation in the cell.

## Introduction

Amyloid-β (Aβ)^3^ peptide is a critical causative constituent of the pathology of Alzheimer's disease (AD) (1) and is known to assemble in multiple configurations that are associated with different physiological or pathological effects (2, 3). Aβ plaques are typically found outside the cell, but considerable recent evidence suggests that intracellular Aβ accumulation also has pathogenic relevance (4). Aβ peptides, most commonly 40 or 42 residues in length, are derived by proteolytic cleavage (5) from amyloid precursor protein (APP), which not only localizes to the plasma membrane but is also found in the trans-Golgi network (6) and multivesicular bodies (7) as well as lysosomal (8) and mitochondrial membranes (9). This provides a substantial source for the production of intracellular aggregation-prone peptide Aβ42 (10–12). It has been shown that in tissues derived from human brain, Aβ42 oligomerization initiates within cells rather than in the extracellular space (13), which may occur by the interaction of Aβ42 with lipid bilayers and lipid rafts (14, 15). In addition, cellular uptake of aggregation-prone peptides via endocytosis also leads to rapid intracellular aggregation (16, 17). This is further supported by a genome-wide study, which revealed that phosphatidylinositol-binding clathrin assembly protein (PICALM) is associated with AD (18, 19), highlighting the significance of endocytic pathways in disease development.

The intracellular aggregation of Aβ42 is an early event in the progression of the neuropathological phenotype, preceding the accumulation of extracellular Aβ42 plaques (20). Furthermore, intracellular Aβ42 impairs synaptic transmission (21), triggers mitochondrial defects (22), and leads to neuronal death. Aβ42 released from dying cells can contribute to extracellular deposits of β-amyloid as the disease progresses (23, 24). Therefore, the study of intracellular Aβ42 accumulation and aggregation is clearly of significance in understanding the development of AD.

Genetic studies of AD have revealed several mutations that aggravate the pathological effects of Aβ42. Among these, the Arctic mutation (APP E693G; Aβ42 E22G) (25) facilitates amyloidosis by early accumulation of intracellular Aβ42 aggregates and a rapid onset of plaque deposition (26). This mutant form of Aβ42 is primarily processed at intracellular locations and forms aggregates inside the cell (27), but there is little information about the structural development or heterogeneity of these aggregates in live cell models. Therefore, an examination of the various states of Arctic mutant aggregation should provide significant insights into the understanding of intracellular amyloidogenesis. In the present study, we constructed inducible, stable, and single-copy cell lines expressing the Arctic mutant form of Aβ42 fused to the fluorescent reporter protein mCherry and carried out direct, in-cell observation of Aβ42 aggregation kinetics using FLIM (16) and super-resolution imaging (28–30). We identified five categories of intracellular aggregate—oligomers, single fibrils, fibril bundles, clusters, and aggresomes—that underline the heterogeneity of Aβ42 aggregates and represent the progression of Aβ42 aggregation within the cell. We also found that Arctic mutant aggregates form more rapidly and to a far greater extent in cells than WT aggregates and are inefficiently degraded.

## Results

### Aβ42 Arctic mutant in cell model displays a fast and aggressive aggregation phenotype

**Figure 1.**
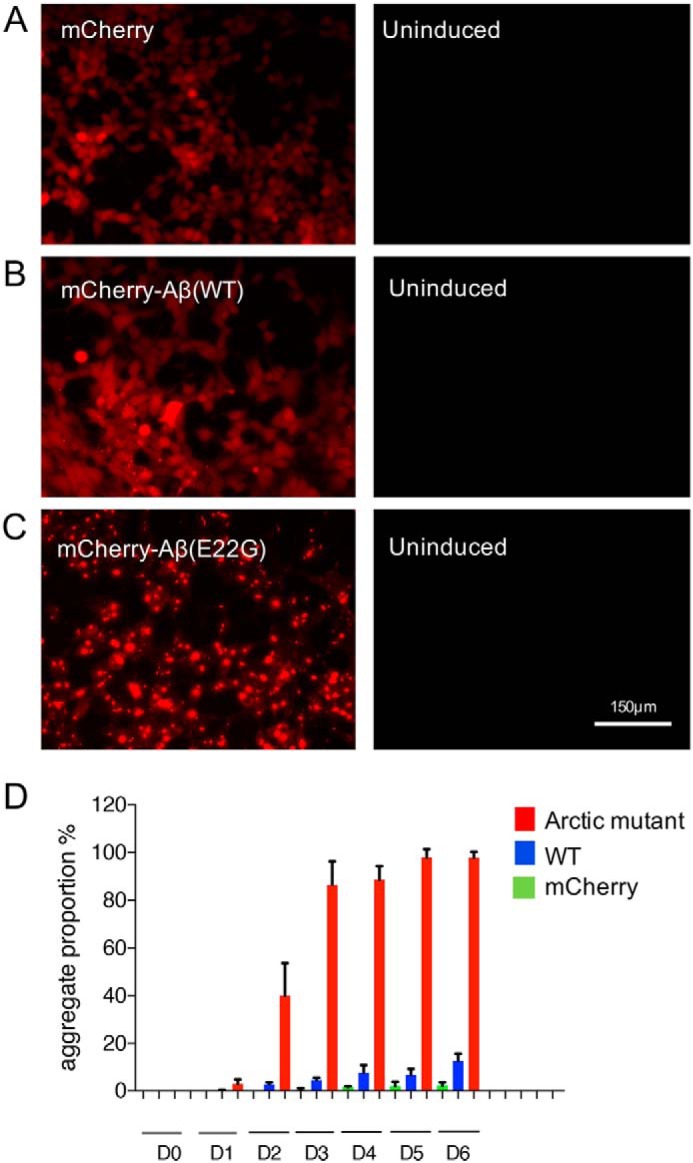
**Arctic mutant expression leads to rapid protein aggregation.** *A*, cells expressing mCherry show a homogeneous distribution of soluble reporter protein. *B*, cells expressing mCherry-Aβ42(WT) generate intracellular aggregates but only in a small proportion of cells. *C*, cells expressing mCherry-Aβ42(E22G) show the development of aggregates in most cells. *D*, proportion of cells containing aggregates, assessed by wide-field microscopy, at different time points after tetracycline induction of gene expression. Three independent experiments were performed with around 200 cells assessed per population per experiment. *D0*, day 0; *D1*, day 1 (24 h after gene induction), etc. *Error bars* represent S.D.

We constructed single-copy, stable cell lines in Flp-In T-REx293 cells with a cytomegalovirus promoter driving mCherry, mCherry-Aβ42(WT), or mCherry-Aβ42(E22G) expression (Fig. S1). Characterization by wide-field fluorescence microscopy showed prominent expression of the reporter proteins in these cell lines (Fig. 1, *A–C*). However, there was a marked difference among them with respect to the amount of protein aggregation. After a week of induction by tetracycline, mCherry was homogeneously distributed in cells (Fig. 1*A*), whereas mCherry-Aβ42 and its Arctic mutant formed aggregates inside cells (Fig. 1, *B* and *C*). Cells expressing the Arctic mutant Aβ42 displayed aggressive aggregation of the fusion protein with very few or no coexisting soluble fragments, indicating that most of the expressed protein is recruited into aggregates (Fig. 1*C*). Such aggressive aggregation was not observed in the mCherry-Aβ42(WT) cell line. In a time course experiment, visible mCherry-Aβ42(E22G) aggregates began to accumulate 24 h after gene induction and within 3 days were present in most cells. However, in the mCherry-Aβ42(WT)–expressing line, cells containing microscopically visible aggregates became apparent more slowly and by the end of the time course were only present in ∼20% of the cell population (Fig. 1*D*). Therefore, our stable cell lines seem to mimic, albeit on a different timescale, the different assembly and aggregation properties of WT and Arctic mutant forms of Aβ42 in the respective forms of AD.

### FLIM reveals different conformation states of in-cell Aβ42 species

**Figure 2.**
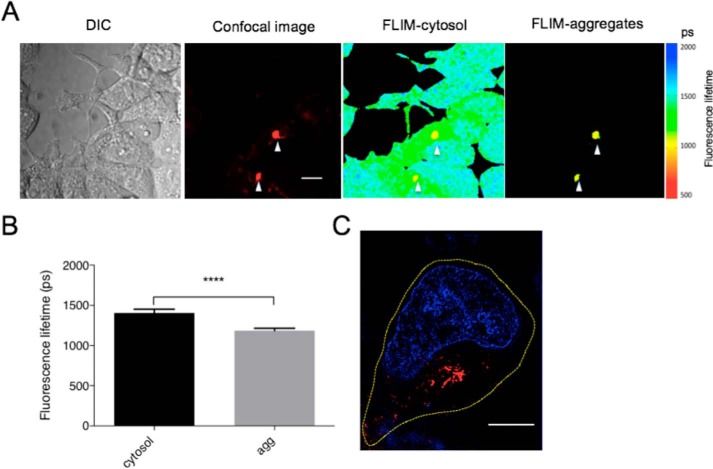
**FLIM reveals different aggregation states of Arctic Aβ42 mutant protein.** *A*, Arctic mutant–expressing cells were induced for 48 h. The *left panel* shows fluorescence intensity of the samples by confocal microscopy, and the *middle panel* shows the fluorescence lifetime of whole cells by single-photon-count FLIM. The *right panel* shows the fluorescence lifetime of aggregates indicated by *white arrows* in the *left panel. Scale bar*, 10 μm. *DIC*, differential interference contrast. *B*, mean fluorescence lifetime of intracellular Arctic mutant fragments in cytosol determined from aggregates (*agg*) in multiple images (*n* = 9). *Error bars* correspond to S.D. **** indicates a *p* value <0.0001 in Student's *t* test. *C*, a representative cell containing aggregates imaged by SIM 48 h after induction. The nucleus was stained with Hoechst 33342 (*blue*). The *yellow dashed line* shows the outline of the cell. *Scale bar*, 5 μm.

To characterize the nature and dynamics of Arctic mutant Aβ42 assembly in cells, we used FLIM (31) to monitor the conversion of soluble protein fragments into amyloid fibrils. In our previous study, a decrease in fluorescence lifetime of a linked fluorophore was correlated with the development of β-sheet–rich amyloid structures (3, 30). In the current study, we first investigated different states of aggregated species by measuring fluorescence decay times of an associated reporter protein, mCherry. After 48 h of gene expression, the fluorescence lifetime of most of the mCherry-Aβ42(E22G) protein within the cell varied little throughout the cytosol, indicating a relatively homogeneous population of Aβ42 species within cells (Fig. 2*A*). Where aggregates were clearly visible, however, a lower fluorescence lifetime was observed (Fig. 2, *A* and *B*), consistent with the conversion of soluble species or loose fibrils to more compacted structures (32). To correlate the fluorescence lifetime with the underlying amyloid structure, we next imaged the same cells by SIM (Fig. 2*C*). In agreement with the fluorescence lifetime results, we showed that the cytosol contained primarily soluble protein, confirming that Aβ42 species were either in the soluble state or associated with a single large perinuclear aggregate, which are the large, and thus visible by confocal microscopy, fibrils that also display the low fluorescence lifetime. The higher resolution of SIM demonstrated this large aggregate to be composed of multiple fibrils.

### Structural progression of Aβ42 Arctic mutant aggregates

**Figure 3.**
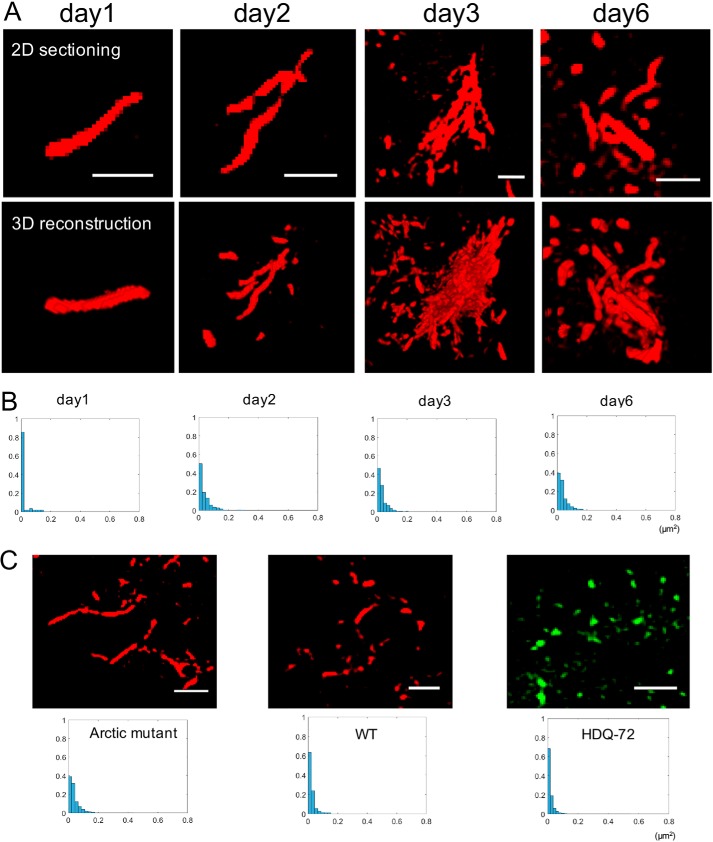
**Structural progression of intracellular Arctic mutant aggregates.** *A*, 3D-SIM demonstrates representative Arctic mutant fibrils and fibril bundles formed inside cells at different times after tetracycline induction. *Upper series of panels*, 2D sectioning slice of the aggregates; *lower series of panels*, projected view from a 3D rendering of the above. *Scale bar*, 1 μm. *B*, size distribution profile of aggregates at different time points. Aggregates from at least three images of each time point were analyzed. *y axis*, proportion; *x axis*, aggregate size (μm^2^). *C*, morphology and size comparison of aggregates formed by three different aggregation-prone proteins: mCherry-Aβ42(E22G), mCherry-Aβ42(WT), and HDQ72-EGFP. Aggregates from at least three images of each cell line were analyzed. *Upper panel*, sectioning slice of aggregates by SIM. *Lower panel*, size distribution profile of aggregates at the same time point (6 days after gene induction).

The structural heterogeneity of the Arctic mutant aggregates suggested by FLIM and 2D SIM (Fig. 2) was next examined using 3D SIM, which can reveal morphological and structural details of aggregated protein species (4). The projection view of 3D SIM was reconstructed from a series of 2D SIM sectioning by Fiji. Fig. 3*A* shows representative fibrillary structures of intracellular Arctic mutant proteins at different stages of maturation. 2D SIM images and the projection view of 3D reconstructions show that, within 24 h of gene induction, Arctic mutant monomers nucleated to form single fibrils of ∼100 nm in diameter and up to ∼2 μm in length (Fig. 3*A*, *day 1*). As more fibrils formed in the cell, they started to assemble into loose clusters in which some fibrils were aligned with each other and apparently cross-linked but also with many gaps in the structure (Fig. 3*A*, *day 2*). The continued accumulation of fibrils gradually led to the formation of bundles, ∼5 μm in diameter, consisting of multiple linear fibrils aligned in a similar orientation (Fig. 3*A*, *day 3*). Although there were still gaps between fibrils, as shown in the 2D section slice, the projection view of its 3D reconstruction showed a dense structure, suggesting the compaction of multiple layers of fibrils. These compact structures were widely observed throughout cells after 3 days of gene induction. By day 6, the fibril assemblies had matured further and showed a very different morphology: the fibril clusters were no longer largely aligned but displayed multiple, tangled branches oriented in various directions. Next, we quantified the size distribution of Arctic mutant aggregates over the 6 days of the experiment by analyzing the 2D SIM images (Fig. 3*B*). On day 1, ∼90% of the amyloid aggregates appeared to have an area of less than 0.02 μm^2^, which is of the order expected for oligomers. Fibrils are greater than 0.02 μm^2^ in size, whereas fibril bundles and clusters exceed 0.1 μm^2^. As the experiment continued, there was a progressive drop in the proportion of oligomers in the overall population from ∼90% (day 1) to less than 40% (day 6), probably reflecting the assembly of oligomeric species into fibrils, fibril bundles, and larger clusters. Thus, fibrils and higher-order assemblies derived from them become the predominant species as the amyloid structures mature.

We further compared the intracellular size distribution of aggregate species of the Aβ42 Arctic mutant with two other aggregation-prone proteins, mCherry-Aβ42(WT) and polyglutamine-containing huntingtin (HDQ72) tagged with GFP (Fig. 3*C*) (33). Six days after induction, the Arctic mutant had the lowest level of oligomeric species (*i.e.* 40%; see above) and the majority of its aggregates in the form of fibrils and related structures, showing it to be the most aggressively aggregation-prone of the three proteins. In comparison, ∼60% of mCherry-Aβ42(WT) aggregates and ∼70% of HDQ72-EGFP aggregates had an area of less than 0.02 μm^2^, *i.e.* were in the form of oligomeric species. This demonstrates that the nature of the aggregation-prone protein, even a difference of one amino acid, can dramatically affect its aggregation properties, specifically in terms of dynamic assembly and structural organization. This highlights the importance of assessing aggregation-prone proteins individually rather than assuming that all such proteins follow the same pattern of aggregation and supramolecular assembly.

### Heterogeneous state of Arctic mutant aggregates inside the cell

**Figure 4.**
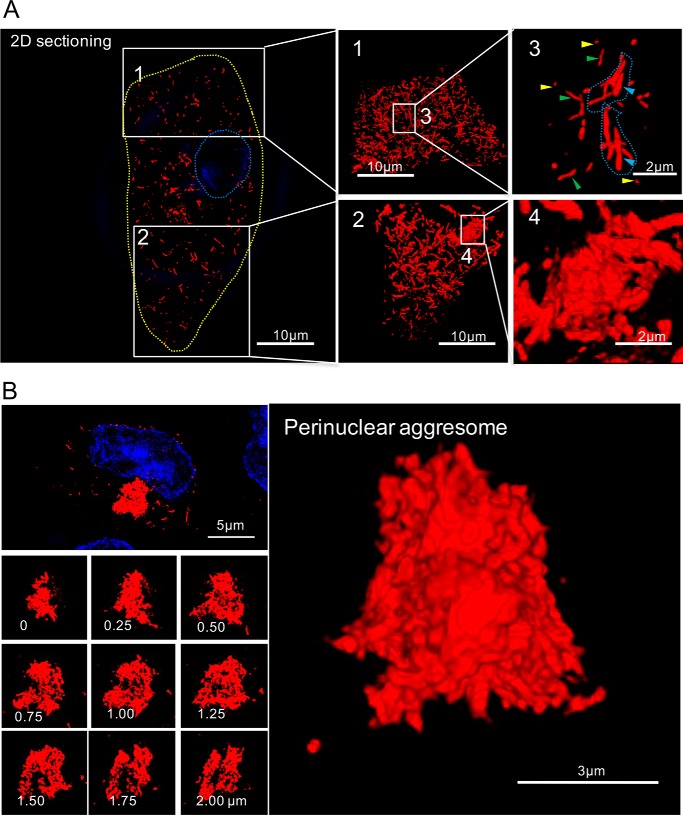
**Super-resolved structures of intracellular Arctic mutant aggregates.** *A*, *left*, a section slice showing an Arctic mutant–expressing cell containing numerous fibrillary fragments, which are in different states of aggregation in the cytosol. The nucleus was stained with Hoechst 33342 (*blue* and *outlined* with a *blue dashed line*). The *yellow dashed line* shows the outline of the cell. The zoomed regions are numbered *1–4. Middle* and *right panels* are the projected views from 3D rendering. *Image 3* shows oligomers (*yellow arrows*), single fibrils (*green arrows*), and fibril bundles (*blue arrows*). *Image 4* shows a fibril cluster. *B*, high-resolution SIM images of an aggresome. The nucleus was stained with Hoechst 33342 (*blue*). *Left panel*, z-stack sectioning slice of an aggresome that contains tightly connected fibrillary fragments. *Right panel*, projected view from 3D rendering of the images from the *left panel*.

We next applied 3D SIM to a more detailed investigation of the different types of mCherry-Aβ42(E22G) aggregate in cells at day 7 postinduction. Because gene expression was continuous throughout this period, the whole range of aggregate species, including oligomers, single fibrils, fibril bundles, fibril clusters, and aggresomes, was present and widely distributed throughout the cytosol in essentially all cells (Fig. 4*A*, *left* and *middle panels*) in which the aggregate species were much more abundant compared with cells at day 2 postinduction (Fig. 2). Compared with 2D section slices (Fig. 4*A*, *left panel*), which show only a single layer of the fibrillar structure, full 3D reconstructions (Fig. 4*A*, *middle panels*) revealed the high density of the aggregate species, mostly fibrils, in this Arctic Aβ42 mutant–expressing cell. This demonstrates the ability of 3D SIM to provide comprehensive details of volumetric structures in cells. The zoomed-in regions in Fig. 4*A* show the heterogeneity of aggregate structures (Video S1 for *image 1* and Video S2 for *image 3*), including oligomers (*image 3*, *yellow arrows*), single fibrils (*image 3*, *green arrows*), fibril bundles (*image 3*, *blue arrows*), and a compact fibril cluster (*image 4*) composed of tangled fibrillary fragments in various orientations.

The mature state of protein aggregation in these cells is characterized by the presence of a perinuclear aggresome, which is formed when the protein degradation system is overwhelmed (34). Aggresomes are highly compact structures comprising branched and tangled fibrils positioned at or near the centrosome (35) (Fig. 4*B* and Video S3). A typical example of an mCherry-Aβ42(E22G) aggresome is shown in the *top left panel* of Fig. 4*B* with z-stack section slices at the depths indicated in the *lower left panels* of Fig. 4*B*. These sections demonstrate the internal organization of tangled fibrils in the aggresome, which is further reconstructed as a 3D structure in the *right panel* of Fig. 4*B*. This amorphous morphology is consistent with the random assembly of fibrillar structures, either by diffusion or active transport, rather than a more ordered assembly of monomeric or oligomeric species at a nucleation site.

### Inefficient degradation of Aβ42 Arctic mutant aggregates

The aggressive accumulation of Arctic mutant aggregates in cells could result from either its rapid assembly into fibrils, its resistance to degradation, or a combination of both. The Aβ42 Arctic mutant has been reported to greatly promote the formation of protofibrils/oligomers (25) and can generate fibrils at much lower concentrations and higher rates than WT Aβ42 *in vitro* (36). In the WT peptide, Glu-22 destabilizes the oligomer structure by electrostatic repulsion between adjacent Glu-22 side chains. *In silico* modeling revealed that substituting glycine at this position replaces glutamic acid with an uncharged residue, resulting in higher oligomer stability (37). The lack of a side chain in glycine may also provide greater conformational flexibility and avoid steric interference among Aβ42 peptides (38). Furthermore, glycine is more hydrophobic and α-helix–destabilizing than glutamic acid (39) and thus virtually eliminates the α-helix propensity in the region adjacent to Gly-22, as shown in molecular dynamics simulations (40). Our results are consistent with the above analyses in the literature and a much faster rate of aggregation of the Arctic mutant than WT Aβ42 in living cells.

**Figure 5.**
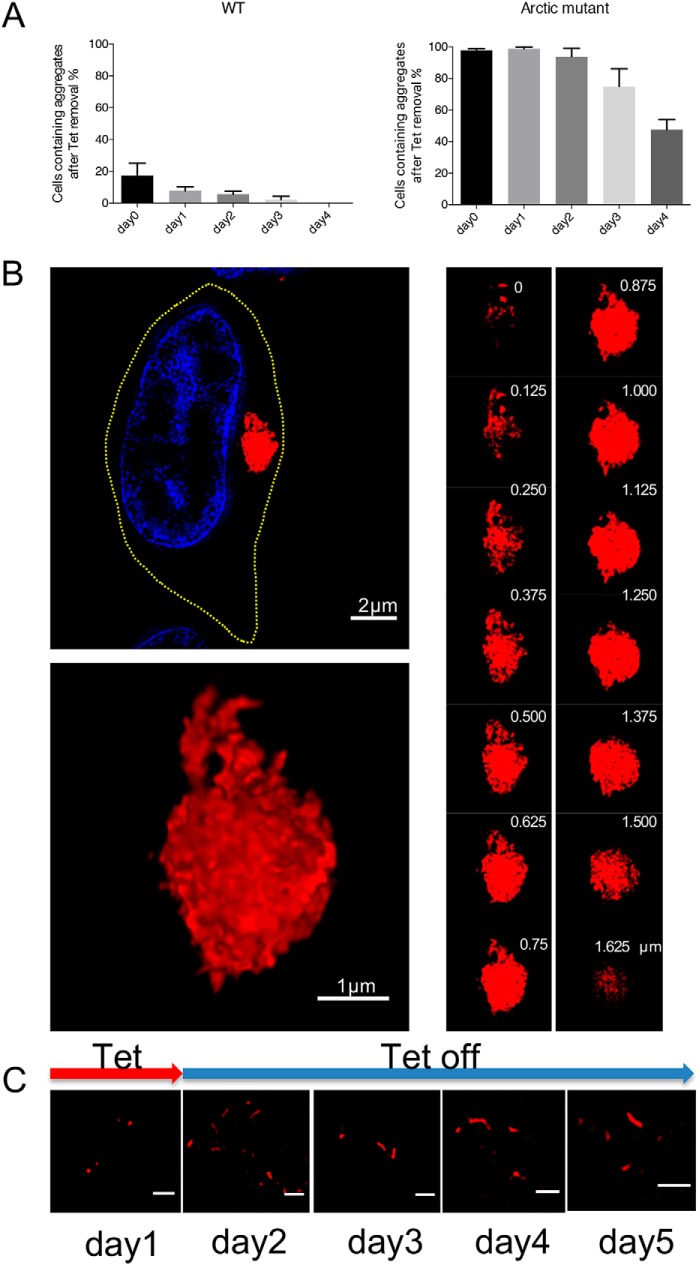
**Arctic mutant aggresomes are resistant to degradation.** *A*, proportion of cells with aggregates (both cytoplasmic aggregates and aggresomes) after the expression of WT or Arctic mutant Aβ42 expression was switched off. Cells were counted at different time points (0, 1, 2, 3, and 4 days) after the medium was replaced with inducer-free medium. Three independent experiments were performed with 200 cells assessed per experiment. *Error bars* represent S.D. *B*, high-resolution SIM images of an aggresome 4 days after gene expression was switched off. *Upper left*, a sectioning slice of a cell containing an aggresome. The nucleus was stained with Hoechst 33342 (*blue*). The *yellow dashed line* shows the cell outline. *Lower left*, projected view from 3D rendering of the aggresome shown above. *Right panel*, z-stack sectioning slice of the same aggresome, which consists of compacted fibrillary fragments. The z-stack depth is labeled in the *upper right* in each image. *C*, 2D-SIM demonstrates persistence of intracellular Arctic mutant fibrils over several days after a short (24-h) pulse of mCherry-Aβ42(E22G) gene expression. The *red arrow* indicates the presence of tetracycline (*Tet*) on day 1 in the culture medium, whereas tetracycline is not present on subsequent days. *Scale bar*, 1 μm.

To test the second premise, that Arctic mutant aggregates are more resistant to degradation than WT, we investigated whether pre-existing mCherry-Aβ42(E22G) aggregates (present after 1 week of induction) can be efficiently degraded when no new Arctic mutant protein is produced. When mCherry-Aβ42(WT) or mCherry-Aβ42(E22G) gene expression was switched off in the respective inducible cell lines, we observed a progressive decrease in the proportion of cells containing aggregates of both types (Fig. 5*A*). After 4 days, the mCherry-Aβ42(WT) cells had degraded almost all of their aggregates; however, ∼50% of the population of Arctic mutant–expressing cells still contained visible aggregates, mainly accumulated in aggresomes, showing that these aggregates are highly resistant to intracellular degradation. To further address this issue, we used 3D SIM to analyze aggregate structures remaining in cells 4 days after mCherry-Aβ42(E22G) gene expression was switched off. As shown in Fig. 5*B*, a representative cell still contained a compact aggresome, whereas separate fibrillar structures were no longer observed, indicating that these smaller species had been cleared from the cytosol. This large aggregate consists of a highly dense core and a less compact peripheral region containing discernible individual species, as shown in the z-section slice (Fig. 5*B*, *right panel*). A large, dense structure of this kind is likely to be more resistant to processing by the ubiquitin-proteasome system and autophagy than looser, smaller assemblies of aggregated protein (41, 42).

Because the Arctic mutant cells generate more aggregates than WT Aβ42 cells, the apparent resistance to degradation of the former aggregates could simply be due to the degradation machinery becoming overwhelmed. To assess this, we incubated mCherry-Aβ42(E22G) cells with tetracycline for 24 h, which resulted in the formation of a limited number of small aggregates and single fibrils (Fig. 5*C*, *day 1*). Then we removed the inducer and analyzed the aggregates in these samples over the following 4 days (Fig. 5*C*, *days 2–5*). We found that the fibrils and small aggregates formed within 24 h persist throughout this period. This is consistent with Arctic mutant aggregates being intrinsically highly resistant to degradation. Thus, the results of Fig. 5*A* are unlikely to be due to the degradation machinery being overwhelmed or to a special property of aggregates within aggresomes.

## Discussion

**Figure 6.**
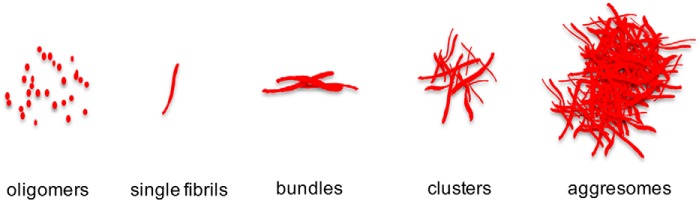
**Five phases in the development of intracellular Aβ42(E22G) aggregates.** The aggregation of Aβ42 Arctic mutant initiates from oligomerization of monomers, which then rapidly assemble to form fibrils. Multiple fibrils connect with high affinity to form bundles, clusters, and eventually aggresomes. The five phases in the structural progression of Arctic mutant aggregates are shown from *left* to *right*. Phase 1, soluble oligomers in cytosol. Phase 2, linear fibrils ranging from 500 nm to 2 μm in length, representing amyloid building blocks and the most universal form of intracellular aggregates. Phase 3, fibril bundles consisting of multiple fibrils aligned in a similar orientation. Phase 4, multiple fibrils assembled as a tightly bound cluster. Phase 5, a large number of fibrillary fragments that form highly compacted aggresomes, which are resistant to degradation.

The emergence and proliferation of Aβ42 plaques in AD brains could be due to several factors: 1) an increase in production of Aβ42 peptides, 2) compromised cellular degradation systems, and/or 3) the rigid structure and stability of Aβ42 amyloid. In this study, we investigated the structural progression of aggregates of the Aβ42 Arctic-mutant peptide in living cells and obtained insights into its fast aggregation and resistance to protein clearance. To do this, we introduced a cell model that expresses the Aβ42 Arctic-mutant peptide, tagged by the fluorescent protein mCherry, which mimics intracellular amyloid formation. Our model demonstrates that a single point mutation in Aβ42, E22G, has a dramatic effect on Aβ42 protein homeostasis, leading to rapid and aggressive amyloid fibril formation. The reporter protein mCherry is shown here to be an excellent marker for fluorescence imaging, including FLIM, which allows quantitative study of the kinetics of amyloidogenesis, and 3D SIM, which enables us to dissect the morphological details of amyloid species at different stages of development at a resolution of 100 nm. The mCherry tagged to Aβ42 could potentially affect the structural progression of aggregates, but other studies (16, 32) have shown that similar fluorescent protein tags take up a peripheral location in developing amyloid and are thus thought to exert a minimal influence on the aggregation process. Because oligomers are normally smaller than 100 nm, SIM would not allow us to examine their morphology in detail. However, they are clearly much shorter than single fibrils, as shown in Fig. 4*A*. Therefore, in this study, we characterized five phases of amyloid development ranging from oligomers to single fibrils, fibril bundles, clusters, and aggresomes (Fig. 6).

We found that, 24 h after mCherry-Aβ42(E22G) gene expression, most of the cytoplasm is filled with species displaying low fluorescence intensity and high lifetime, suggesting a soluble pool of Arctic mutant throughout the cytoplasm, whereas the intracellular regions with higher intensity and lower lifetime may reflect the rapid accumulation of oligomers or single fibrils (43). The formation of aggresomes with prominent fluorescence intensity led to a dramatic decrease in fluorescence lifetime, which is an indication of highly compacted structure via the mechanism of mCherry self-quenching (44). This demonstrates the highly compact structures of aggresomes, as further revealed by 3D SIM (Fig. 4). We also characterized the structural progression of amyloid, primarily for the size and morphology of different aggregation states. The 3D structures reconstructed from SIM provide us with an unprecedented volumetric view of aggregates proliferating in the whole cell (Figs. 3 and 4), which provides a more comprehensive model of amyloid fibril organization, distribution, and structures.

In the development of aggregates, we found that fibrils first aligned in the same orientation to form fibril bundles and then assembled in a random way to form clusters and aggresomes as the intracellular fibril concentration increases. We demonstrated that the single residue change in the mutant Aβ42 peptide leads to significant differences in amyloid progression and structures, which are resistant to intracellular degradation. How these different amyloid states lead to intracellular toxicity needs to be fully addressed in the future; this model provides a platform for studying the effect of intracellular amyloid on the function of various cellular compartments. In addition, because this model demonstrates various stages of protein aggregation, it can also be used for the screening of antiaggregation compounds.

## Experimental procedures

### Cells

Sequence of plasmids used for stable cell line construction are given in the supporting information. Mammalian Flp-In T-REx293 cells were grown in T75 or T25 flasks or 6-well plates by incubation at 37 °C in a 5% CO_2_ atmosphere (35). Complete medium consists of 90% Dulbecco's modified Eagle's medium, 10% fetal bovine serum, and 2 mm l-glutamine; antibiotics were used as appropriate (35). Cells were passaged on reaching 80–90% confluence (approximately every 3–4 days) and kept in logarithmic phase growth. Routine cell counting and viability assays were carried out using a hemocytometer and trypan blue. Transfections were performed on cells at 80% confluence. Stable cell line construction has been described previously (32).

### Microscopy

The protocol of cell fixation for imaging was as described previously (32). After induction for various times, cells were fixed, and images were recorded with an OMX V3 super-resolution microscope (35).

Confocal microscopy was performed as described previously (35). FLIM experiments were carried out using custom-built time-correlated single photon counting as described before (16). FLIM images were analyzed by FLIMfit (31). Imaging of section slices was acquired from the OMX, and 3D reconstruction from multiple section slices was performed by Fiji Volume Viewer, which produced 3D projection views of the reconstruction.

## Author contributions

M. L., N. W., A. T., and G. S. K. S. conceptualization; M. L., N. W., A. T., and G. S. K. S. resources; M. L., A. T., and G. S. K. S. data curation; M. L., C. H. M., A. T., and G. S. K. S. formal analysis; M. L. validation; M. L., N. W., A. M., and C. H. M. investigation; M. L., C. F. K., A. T., and G. S. K. S. methodology; M. L., A. T., and G. S. K. S. writing-original draft; M. L., A. M., C. F. K., A. T., and G. S. K. S. writing-review and editing; C. F. K., A. T., and G. S. K. S. supervision; C. F. K., A. T., and G. S. K. S. funding acquisition; A. T. and G. S. K. S. project administration.

## Supplementary Material

Supporting Information

## Footnotes

3 The abbreviations used are:

Aβ: amyloid-β
FLIM: fluorescence-lifetime imaging
SIM: structural illumination microscopy
AD: Alzheimer's disease
APP: amyloid precursor protein
EGFP: enhanced GFP.

## References

[1] Selkoe D. J., and Hardy J. (2016) The amyloid hypothesis of Alzheimer's disease at 25 years. EMBO Mol. Med. 8, 595–608 10.15252/emmm.201606210 27025652 PMC4888851

[2] Murphy M. P., and Levine H. 3rd (2010) Alzheimer's disease and the amyloid-β peptide. J. Alzheimers Dis. 19, 311–323 10.3233/JAD-2010-1221 20061647 PMC2813509

[3] Eisenberg D., and Jucker M. (2012) The amyloid state of proteins in human diseases. Cell 148, 1188–1203 10.1016/j.cell.2012.02.022 22424229 PMC3353745

[4] LaFerla F. M., Green K. N., and Oddo S. (2007) Intracellular amyloid-β in Alzheimer's disease. Nat. Rev. Neurosci. 8, 499–509 10.1038/nrn2168 17551515

[5] Haass C., Kaether C., Thinakaran G., and Sisodia S. (2012) Trafficking and proteolytic processing of APP. (2012) Cold Spring Harb. Perspect. Med. 2, a006270 10.1101/cshperspect.a006270 22553493 PMC3331683

[6] Xu H., Greengard P., and Gandy S. (1995) Regulated formation of Golgi secretory vesicles containing Alzheimer β-amyloid precursor protein. J. Biol. Chem. 270, 23243–23245 10.1074/jbc.270.40.23243 7559474

[7] Morel E., Chamoun Z., Lasiecka Z. M., Chan R. B., Williamson R. L., Vetanovetz C., Dall'Armi C., Simoes S., Point Du Jour K. S., McCabe B. D., Small S. A., and Di Paolo G. (2013) Phosphatidylinositol-3-phosphate regulates sorting and processing of amyloid precursor protein through the endosomal system. Nat. Commun. 4, 2250 10.1038/ncomms3250 23907271 PMC3905799

[8] Kinoshita A. (2003) Demonstration by FRET of BACE interaction with the amyloid precursor protein at the cell surface and in early endosomes. J. Cell Sci. 116, 3339–3346 10.1242/jcs.00643 12829747

[9] Mizuguchi M., Ikeda K., and Kim S. U. (1992) Differential distribution of cellular forms of β-amyloid precursor protein in murine glial cell cultures. Brain Res. 584, 219–225 10.1016/0006-8993(92)90898-J 1515940

[10] Wertkin A. M., Turner R. S., Pleasure S. J., Golde T. E., Younkin S. G., Trojanowski J. Q., and Lee V. M. (1993) Human neurons derived from a teratocarcinoma cell line express solely the 695-amino acid amyloid precursor protein and produce intracellular β-amyloid or A4 peptides. Proc. Natl. Acad. Sci. U.S.A. 90, 9513–9517 10.1073/pnas.90.20.9513 8415732 PMC47599

[11] Rovelet-Lecrux A., Hannequin D., Raux G., Le Meur N., Laquerrière A., Vital A., Dumanchin C., Feuillette S., Brice A., Vercelletto M., Dubas F., Frebourg T., and Campion D. (2006) APP locus duplication causes autosomal dominant early-onset Alzheimer disease with cerebral amyloid angiopathy. Nat. Genet. 38, 24–26 10.1038/ng1718 16369530

[12] Schreiner B., Hedskog L., Wiehager B., and Ankarcrona M. (2015) Amyloid-β peptides are generated in mitochondria-associated endoplasmic reticulum membranes. J. Alzheimers Dis. 43, 369–374 10.3233/JAD-132543 25096627

[13] Walsh D. M., Tseng B. P., Rydel R. E., Podlisny M. B., and Selkoe D. J. (2000) The oligomerization of amyloid β-protein begins intracellularly in cells derived from human brain. Biochemistry 39, 10831–10839 10.1021/bi001048s 10978169

[14] Kawarabayashi T., Shoji M., Younkin L. H., Wen-Lang L., Dickson D. W., Murakami T., Matsubara E., Abe K., Ashe K. H., and Younkin S. G. (2004) Dimeric amyloid β protein rapidly accumulates in lipid rafts followed by apolipoprotein E and phosphorylated tau accumulation in the Tg2576 mouse model of Alzheimer's disease. J. Neurosci. 24, 3801–3809 10.1523/JNEUROSCI.5543-03.2004 15084661 PMC6729359

[15] Kim S. I., Yi J. S., and Ko Y. G. (2006) Amyloid β oligomerization is induced by brain lipid rafts. J. Cell. Biochem. 99, 878–889 10.1002/jcb.20978 16721824

[16] Esbjörner E. K., Chan F., Rees E., Erdelyi M., Luheshi L. M., Bertoncini C. W., Kaminski C. F., Dobson C. M., and Kaminski Schierle G. S. (2014) Direct observations of amyloid β self-assembly in live cells provide insights into differences in the kinetics of Aβ(1–40) and Aβ(1–42) aggregation. Chem. Biol. 21, 732–742 10.1016/j.chembiol.2014.03.014 24856820 PMC4067742

[17] Michel C. H., Kumar S., Pinotsi D., Tunnacliffe A., St George-Hyslop P., Mandelkow E., Mandelkow E. M., Kaminski C. F., and Schierle G. S. (2014) Extracellular monomeric tau protein is sufficient to initiate the spread of tau protein pathology. J. Biol. Chem. 289, 956–967 10.1074/jbc.M113.515445 24235150 PMC3887218

[18] Harold D., Abraham R., Hollingworth P., Sims R., Gerrish A., Hamshere M. L., Pahwa J. S., Moskvina V., Dowzell K., Williams A., Jones N., Thomas C., Stretton A., Morgan A. R., Lovestone S., et al (2009) Genome-wide association study identifies variants at CLU and PICALM associated with Alzheimer's disease. Nat. Genet. 41, 1088–1093 10.1038/ng.440 19734902 PMC2845877

[19] Zhao Z., Sagare A. P., Ma Q., Halliday M. R., Kong P., Kisler K., Winkler E. A., Ramanathan A., Kanekiyo T., Bu G., Owens N. C., Rege S. V., Si G., Ahuja A., Zhu D., et al (2015) Central role for PICALM in amyloid-β blood-brain barrier transcytosis and clearance. Nat. Neurosci. 18, 978–987 10.1038/nn.4025 26005850 PMC4482781

[20] Oddo S., Caccamo A., Smith I. F., Green K. N., and LaFerla F. M. (2006) A dynamic relationship between intracellular and extracellular pools of Aβ42. Am. J. Pathol. 168, 184–194 10.2353/ajpath.2006.050593 16400022 PMC1592652

[21] Ripoli C., Cocco S., Li Puma D. D., Piacentini R., Mastrodonato A., Scala F., Puzzo D., D'Ascenzo M., and Grassi C. (2014) Intracellular accumulation of amyloid-β (Aβ42) protein plays a major role in Aβ42-induced alterations of glutamatergic synaptic transmission and plasticity. J. Neurosci. 34, 12893–12903 10.1523/JNEUROSCI.1201-14.2014 25232124 PMC6705320

[22] Schaefer P. M., von Einem B., Walther P., Calzia E., and von Arnim C. A. (2016) Metabolic characterization of intact cells reveals intracellular amyloid γ but not its precursor protein to reduce mitochondrial respiration. PLoS One 11, e0168157 10.1371/journal.pone.0168157 28005987 PMC5178995

[23] D'Andrea M. R., Nagele R. G., Wang H. Y., Peterson P. A., and Lee D. H. (2001) Evidence that neurones accumulating amyloid can undergo lysis to form amyloid plaques in Alzheimer's disease. Histopathology 38, 120–134 10.1046/j.1365-2559.2001.01082.x 11207825

[24] Pensalfini A., Albay R. 3rd, Rasool S., Wu J. W., Hatami A., Arai H., Margol L., Milton S., Poon W. W., Corrada M. M., Kawas C. H., and Glabe C. G. (2014) Intracellular amyloid and the neuronal origin of Alzheimer neuritic plaques. Neurobiol. Dis. 71, 53–61 10.1016/j.nbd.2014.07.011 25092575 PMC4179983

[25] Nilsberth C., Westlind-Danielsson A., Eckman C. B., Condron M. M., Axelman K., Forsell C., Stenh C., Luthman J., Teplow D. B., Younkin S. G., Näslund J., and Lannfelt L. (2001) The “Arctic” APP mutation (E693G) causes Alzheimer's disease by enhanced Aβ42 protofibril formation. Nat. Neurosci. 4, 887–893 10.1038/nn0901-887 11528419

[26] Lord A., Kalimo H., Eckman C., Zhang X. Q., Lannfelt L., and Nilsson L. N. (2006) The Arctic Alzheimer mutation facilitates early intraneuronal Aβ42 aggregation and senile plaque formation in transgenic mice. Neurobiol. Aging 27, 67–77 10.1016/j.neurobiolaging.2004.12.007 16298242

[27] Knobloch M., Konietzko U., Krebs D. C., and Nitsch R. M. (2007) Intracellular Aβ and cognitive deficits precede β-amyloid deposition in transgenic ArcAβ mice. Neurobiol. Aging 28, 1297–1306 10.1016/j.neurobiolaging.2006.06.019 16876915

[28] Young L., Ströhl F., and Kaminski C. (2016) A guide to structured illumination TIRF microscopy at high speed with multiple colors. J. Vis. Exp. e53988 10.3791/53988 27285848 PMC4927749

[29] Pinotsi D., Buell A. K., Galvagnion C., Dobson C. M., Kaminski Schierle G. S., and Kaminski C. F. (2014) Direct observation of heterogeneous amyloid fibril growth kinetics via two-color super-resolution microscopy. Nano Lett. 14, 339–345 10.1021/nl4041093 24303845 PMC3901574

[30] Kaminski Schierle G. S., van de Linde S., Erdelyi M., Esbjörner E. K., Klein T., Rees E., Bertoncini C. W., Dobson C. M., Sauer M., and Kaminski C. F. (2011) *In situ* measurements of the formation and morphology of intracellular β-amyloid fibrils by super-resolution fluorescence imaging. J. Am. Chem. Soc. 133, 12902–12905 10.1021/ja201651w 21793568

[31] Warren S. C., Margineanu A., Alibhai D., Kelly D. J., Talbot C., Alexandrov Y., Munro I., Katan M., Dunsby C., and French P. M. (2013) Rapid global fitting of large fluorescence lifetime imaging microscopy datasets. PLoS One 8, e70687 10.1371/journal.pone.0070687 23940626 PMC3734241

[32] Chen W., Young L. J., Lu M., Zaccone A., Ströhl F., Yu N., Kaminski Schierle G. S., and Kaminski C. F. (2017) Fluorescence self-quenching from reporter dyes informs on the structural properties of amyloid clusters formed *in vitro* and in cells. Nano Lett. 17, 143–149 10.1021/acs.nanolett.6b03686 28073262 PMC5338000

[33] Lu M., Williamson N., Boschetti C., Ellis T., Yoshimi T., and Tunnacliffe A. (2015) Expression-level dependent perturbation of cell proteostasis and nuclear morphology by aggregation-prone polyglutamine proteins. Biotechnol. Bioeng. 112, 1883–1892 10.1002/bit.25606 25854808

[34] Johnston J. A., Ward C. L., and Kopito R. R. (1998) Aggresomes: a cellular response to misfolded proteins. J. Cell Biol. 143, 1883–1898 10.1083/jcb.143.7.1883 9864362 PMC2175217

[35] Lu M., Boschetti C., and Tunnacliffe A. (2015) Long term aggresome accumulation leads to DNA damage, p53-dependent cell cycle arrest, and steric interference in mitosis. J. Biol. Chem. 290, 27986–28000 10.1074/jbc.M115.676437 26408200 PMC4646037

[36] Norlin N., Hellberg M., Filippov A., Sousa A. A., Gröbner G., Leapman R. D., Almqvist N., and Antzutkin O. N. (2012) Aggregation and fibril morphology of the Arctic mutation of Alzheimer's Aβ42 peptide by CD, TEM, STEM and *in situ* AFM. J. Struct. Biol. 180, 174–189 10.1016/j.jsb.2012.06.010 22750418 PMC3466396

[37] Kassler K., Horn A. H., and Sticht H. (2010) Effect of pathogenic mutations on the structure and dynamics of Alzheimer's Aβ42-amyloid oligomers. J. Mol. Model. 16, 1011–1020 10.1007/s00894-009-0611-1 19908073

[38] Perálvarez-Marín A., Mateos L., Zhang C., Singh S., Cedazo-Mínguez A., Visa N., Morozova-Roche L., Gräslund A., and Barth A. (2009) Influence of residue 22 on the folding, aggregation profile, and toxicity of the Alzheimer's amyloid β peptide. Biophys. J. 97, 277–285 10.1016/j.bpj.2009.04.017 19580765 PMC2711388

[39] O'Neil K. T., and DeGrado W. F. (1990) A thermodynamic scale for the helix-forming tendencies of the commonly occurring amino acids. Science 250, 646–651 10.1126/science.2237415 2237415

[40] Lin Y. S., and Pande V. S. (2012) Effects of familial mutations on the monomer structure of Aβ42. Biophys. J. 103, L47–L49 10.1016/j.bpj.2012.11.009 23260058 PMC3525840

[41] Verhoef L. G., Lindsten K., Masucci M. G., and Dantuma N. P. (2002) Aggregate formation inhibits proteasomal degradation of polyglutamine proteins. Hum. Mol. Genet. 11, 2689–2700 10.1093/hmg/11.22.2689 12374759

[42] Tanik S. A., Schultheiss C. E., Volpicelli-Daley L. A., Brunden K. R., and Lee V. M. (2013) Lewy body-like α-synuclein aggregates resist degradation and impair macroautophagy. J. Biol. Chem. 288, 15194–15210 10.1074/jbc.M113.457408 23532841 PMC3663539

[43] Cohen S. I. A., Linse S., Luheshi L. M., Hellstrand E., White D. A., Rajah L., Otzen D. E., Vendruscolo M., Dobson C. M., and Knowles T. P. (2013) Proliferation of amyloid-β42 aggregates occurs through a secondary nucleation mechanism. Proc. Natl. Acad. Sci. U.S.A. 110, 9758–9763 10.1073/pnas.1218402110 23703910 PMC3683769

[44] Kruitwagen T., Denoth-Lippuner A., Wilkins B. J., Neumann H., and Barral Y. (2015) Axial contraction and short-range compaction of chromatin synergistically promote mitotic chromosome condensation. Elife 4, e1039 10.7554/eLife.10396 26615018 PMC4755758

