# Supplementary material for: Structural progression of amyloid-β Arctic mutant aggregation in cells revealed by multiparametric imaging
Source: J Biol Chem. 2018 Nov 30;294(5):1478–87. doi: 10.1074/jbc.RA118.004511 (PMC6364760; doi:10.1074/jbc.RA118.004511)
Supplement: Supporting Information [file supp_294_5_1478__index.html]

Structural progression of amyloid-β Arctic mutant aggregation in cells revealed by multi-parametric imaging — Structural progression of amyloid-β Arctic mutant — Structural progression of amyloid-β Arctic mutant aggregation in cells revealed by multiparametric imaging — Structural progression of amyloid-β Arctic mutant — Supporting Information 

# Structural progression of amyloid-β Arctic mutant aggregation in cells revealed by multiparametric imaging

## Supporting Information

- Supporting figures - plasmid constructs
- video1 - 3D SIM reconstruction of fibril pools in cytosol
- video2 - 3D SIM reconstruction of oligomers, single fibrils and fibril clusters
- video3 - 3D SIM reconstruction of a compacted aggresome
